# Nutrient sensors and their crosstalk

**DOI:** 10.1038/s12276-023-01006-z

**Published:** 2023-06-01

**Authors:** Yulseung Sung, Ya Chun Yu, Jung Min Han

**Affiliations:** 1 Yonsei Institute of Pharmaceutical Sciences, College of Pharmacy, Yonsei University, Incheon, 21983 South Korea; 2 Department of Integrated OMICS for Biomedical Science, Yonsei University, Seoul, 03722 South Korea; 3 POSTECH Biotech Center, Pohang University of Science and Technology, Pohang, 37673 South Korea

**Keywords:** Nutrient signalling, Cell growth

## Abstract

The macronutrients glucose, lipids, and amino acids are the major components that maintain life. The ability of cells to sense and respond to fluctuations in these nutrients is a crucial feature for survival. Nutrient-sensing pathways are thus developed to govern cellular energy and metabolic homeostasis and regulate diverse biological processes. Accordingly, perturbations in these sensing pathways are associated with a wide variety of pathologies, especially metabolic diseases. Molecular sensors are the core within these sensing pathways and have a certain degree of specificity and affinity to sense the intracellular fluctuation of each nutrient either by directly binding to that nutrient or indirectly binding to its surrogate molecules. Once the changes in nutrient levels are detected, sensors trigger signaling cascades to fine-tune cellular processes for energy and metabolic homeostasis, for example, by controlling uptake, de novo synthesis or catabolism of that nutrient. In this review, we summarize the major discoveries on nutrient-sensing pathways and explain how those sensors associated with each pathway respond to intracellular nutrient availability and how these mechanisms control metabolic processes. Later, we further discuss the crosstalk between these sensing pathways for each nutrient, which are intertwined to regulate overall intracellular nutrient/metabolic homeostasis.

## Metabolism: nutrient sensors and their networks

The ability of cells to sense levels of important nutrients, such as glucose, lipids, and amino acids, is critical for cellular health, and dysregulation such sensing activity can underlie various diseases, especially metabolic diseases. Sensing nutrient levels allows cells to regulate their metabolism and to maintain appropriate nutrient levels by taking up, synthesizing, or breaking down a given nutrient. Jung Min Han at Yonsei University in South Korea and co-workers have reviewed recent discoveries on nutrient sensors and the networks connecting them. Although researchers have identified the sensors of some key molecules, such as glucose and important amino acids such as leucine, how sensing networks interact is poorly understood. Improving our understanding of nutrient sensing will illuminate nutrient supply in both health and disease, and may help to find treatments for metabolic disorders.

## Introduction

Glucose, amino acids, and lipids are carefully regulated to coordinate different signaling pathways for cellular survival^1–3^. The cautious association of nutrient utilization is therefore crucial for maintaining cellular homeostasis. These types of associations can be organized by cells, tissues, and organisms^4,5^. For example, pancreatic tissue senses the glucose state and secretes different hormones to stimulate the brain and gut to increase or decrease food intake^6^. However, in this review, we will focus on the association of nutrient utilization by the cell. To maintain cellular homeostasis, cells must be able to not only take up nutrients but also to catabolize nutrients. Therefore, cells require the ability to sense different nutrient states to adapt to various environments and increase or decrease nutrient availability. To be able to do so, the intracellular signaling pathways of nutrient sensing are regulated by different sensor proteins. Nutrient sensor proteins can be defined as proteins that are able to directly interact with a certain substrate and develop different outcomes depending on the nutrient concentration. These nutrient sensors are important biological action proteins that manipulate cellular fate. Some are yet to be discovered, but other sensors, such as leucine sensors, have been researched to an extreme level^1,7,8^. In addition, nutrients are all connected. Glucose metabolites react with different enzymes to form scarce lipid resources, and amino acids, such as glutamine, enter the mitochondria to undergo reductive carboxylation to fuel TCA cycle metabolites, which normally originate from glucose^9–11^. Therefore, a large amount of evidence indicates that different nutrients crosstalk with each other to maintain cellular homeostasis. This review will introduce several studies that reveal the crosstalk of different nutrients and suggest potential crosstalk by connecting different studies in the same context.

## Nutrient sensors

### Glucose sensing

**Fig. 1:**
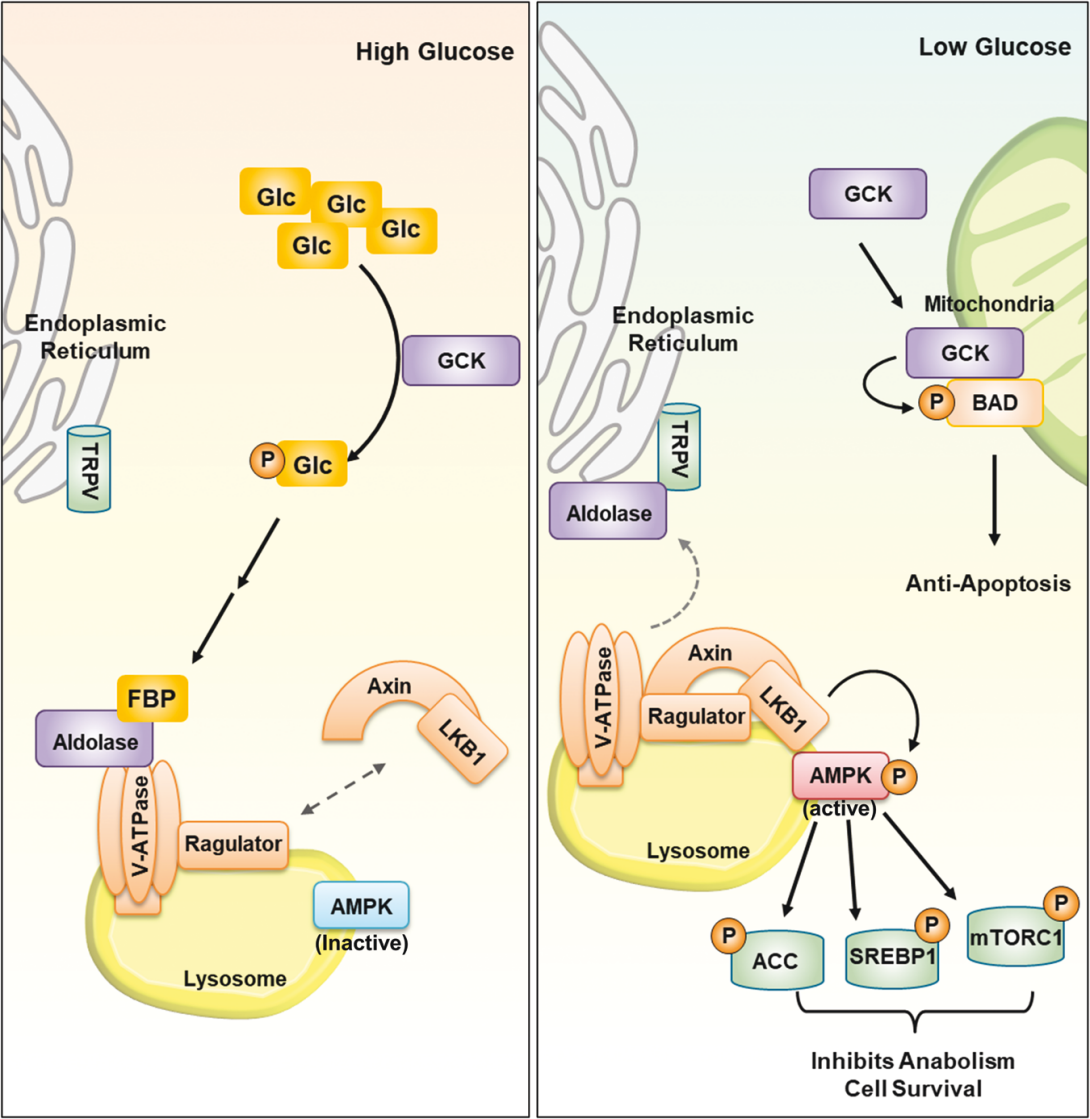
Glucose sensors. Glucose can be sensed by GCK, GLUT2, and aldolase. When glucose is supplemented at a high level, it is phosphorylated by GCK, a hexokinase protein, to produce ATP. Under glucose deprivation, GCK interacts with BAD in the mitochondria. GCK phosphorylates BAD to promote the reduction of apoptosis. GLUT2 takes up glucose only when the glucose level is high. As glucose is imported by GLUT2, GLUT2 will increase the intracellular calcium level to secrete insulin. Under low-glucose conditions, free aldolase proteins localize in the lysosomal membrane, inhibiting TRPV channels and forming a V-ATPase-Ragulator complex stabilized by LKB1-Axin. This complex initiates the activation of AMPK for downstream signaling pathways. Under high-glucose conditions, FBP levels are increased. FBP binds to aldolase, which inhibits its localization and complex formation in the lysosome.

Among the macronutrients that mammals utilize, carbohydrates function to produce energy. An organism breaks down carbohydrates into glucose for cellular uptake, which modulates various cellular signaling pathways. Consequently, maintenance of normal glucose levels is an essential function of cell survival. Glucose sensors are therefore extremely crucial for cellular survival and response to different stimuli. Glucokinase, GLUT2, and aldolase are known glucose sensors (Fig. 1).

#### Glucokinase (GCK)

Although the ratio varies depending on the cell type, intracellular glucose has three fates: (1) oxidative phosphorylation after undergoing glycolysis for energy production, (2) glycogen synthesis as a polysaccharide for storage, and (3) modification into other cellular metabolites for further cellular pathways^12^. The first reaction that glucose undergoes for energy production is conversion into glucose-6-phosphate^13^. This reaction is catalyzed by the hexokinase protein family. As hexokinases directly interact with glucose, they are speculated to be glucose sensors. However, among the hexokinase family, only glucokinase (GCK) has been found to be a glucose sensor^14^. This is because compared with other hexokinase proteins, GCK has a significantly lower affinity for glucose. Unlike GCK, other hexokinases interact with glucose regardless of the intracellular concentration of glucose, signifying that they metabolize glucose regardless of its availability; GCK only phosphorylates glucose when the intracellular glucose level is high. This characteristic is reflected in the Km value: the Km value for other hexokinases is ~0.2 mM, while the Km value for glucokinase is ~8 mM^15^. The outcome of the sensor function of GCK is the regulation of cellular apoptosis. Under high-glucose conditions, GCK binds with glucose and becomes activated. This could lead to the phosphorylation of glucose for ATP production, and GCK would interact with BAD, which is localized in the mitochondrial membrane^16^. Activated GCK protein opens a protein‒protein interaction site with BAD, which results in the phosphorylation of BAD^17^. Phosphorylated BAD promotes the reduction of apoptotic proteins for cellular survival. Under low glucose levels, GCK will be inactivated where the BAD interaction site is hidden, leading to the normal function of BAD in promoting apoptosis. However, there are some controversies regarding GCK as a glucose sensor. Although a much deeper understanding is needed, glucose-sensing defects were not observed in *GCK* mutation carriers^18^.

#### GLUT2

Research on the upstream pathways of GCK was conducted for further clarification. GCK phosphorylates glucose, which is transported by the glucose transporter family. Among the family members, such as GCK, GLUT2 is a glucose transporter with an exceptionally high glucose Km value of 20 mM^19^. Compared with GLUT1 (Km = 1 mM) and GLUT4 (Km = 5 mM), GLUT2 has a lower glucose affinity. Therefore, GLUT2 only becomes active when the glucose concentration is high. Similar to GCK, GLUT2 was studied as a potential glucose sensor. As GLUT2 is a bidirectional carrier protein, it exports glucose when the intracellular glucose level is high, attempting to maintain cellular homeostasis^20^. However, the outcome of GLUT2 glucose-sensing activity is not clear from an intracellular point of view. GLUT2 can control the hyperglycemic state by regulating the ATP:ADP ratio^21^. The import activity of GLUT2 will increase the calcium level, which stimulates the exocytosis of intracellular insulin vacuoles, leading to hormonal consequences. However, the outcome of the glucose-sensing algorithm of GLUT2 is still unknown, challenging the relevance of GLUT2 as a glucose sensor^20^.

#### Aldolase and AMPK

Recently, one group demonstrated that aldolase, a critical enzyme in the glycolysis pathway, is a sensor for both high and low glucose levels^22^. GCK and GLUT2 proteins both showed outcomes when the glucose level was high. However, this group revealed a protein that acts as a reliable glucose sensor because it showed different outcomes depending on the glucose level. Through glycolysis, glucose is catalyzed into fructose-1,6-bisphosphate (FBP), which is the substrate of aldolase. This group showed that a low-glucose state produces a high amount of free aldolase in the cytoplasm and that in a high-glucose state, FBP-bound aldolases exist at high concentrations^23^. These distinct states of aldolases lead to different cellular signaling pathway activation. Briefly, glucose availability directly reflects FBP availability, leading to different aldolase states. The outcome of the sensor function of aldolase is linked to AMPK activation^23^. AMPK is the primary kinase that becomes activated under nutrient stresses, especially glucose deprivation. Previously, AMPK activation was believed to be related to the ATP:ADP ratio^2^. However, in glucose-deprived conditions, aldolase is recruited to the lysosomal membrane along with TRPV channels on the endoplasmic reticulum. Free aldolase inhibits calcium release by TRPV, which becomes accessible for binding to V-ATPase^24^. The aldolase/TRPV/V-ATPase complex inhibits v-ATPase activity and allows the binding of Ragulator via the LKB1-Axin complex^25^. This results in the phosphorylation of LKB1, leading to AMPK activation and resolving nutrient stress via a downstream signaling pathway. On the other hand, upon glucose stimulation, the FBP level increases, which increases the amount of FBP-bound aldolases. Without free aldolases, TRPV channels pass calcium ions freely, and AMPK-activating protein recruitment does not occur^26^. This cellular outcome further confirms that aldolase is a glucose sensor. As aldolase is a critical nutrient sensor, it was targeted for diabetes treatment^27^. rSjcystatin and rSjFBPA, which competitively inhibit aldolase, were developed to improve inflammatory tissue damage due to type 1 diabetes. Aldolase inhibition was also utilized to ammeliorate diabetic retinopathy. Autoantibodies against aldolase were observed at a significantly higher level in patients with lower diabetic retinopathy disease^28^.

### Amino acid sensing

**Fig. 2:**
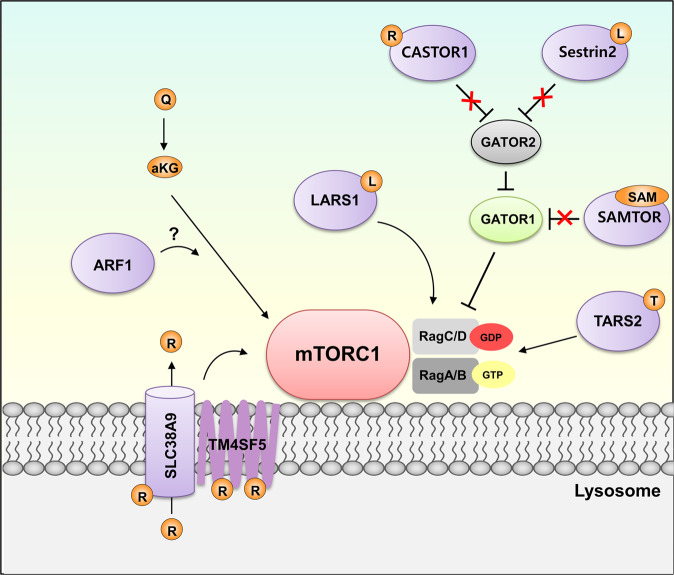
mTORC1-related amino acid sensors. Leucine is sensed by LARS1 and sestrin2. Leucine-bound LARS1 activates mTORC1 through the RagGTP pathway, and leucine-bound sestrin2 inhibits GATOR2, which stimulates the mTORC1 complex. The methionine derivative metabolite S-adenosylmethionine is sensed by SMATOR. SAMTOR binds SAM at the GATOR-KICSTOR binding domain. This will allow GATOR1 to be inactivated and inhibit its GAP activity to stimulate mTORC1 function. Arginine-bound TM4SF5 induces SLC38A9 to pump arginine out of the lysosomal membrane. Increased arginine concentration allows the binding of arginine to CASTOR1, which induces the dissociation of GATOR2 from CASTOR1, increasing the activity of mTORC1. Threonine also becomes charged to TARS2. This protein interacts with RagC and activates GEF function to produce RagA-GTP, which activates the mTORC1 complex. Although the sensor has not yet been discovered, glutamine-derived alpha-ketoglutarate seems to induce mTORC1 activation by Arf1.

Amino acids, the essential building blocks of proteins, are important nutrients for cell growth. In addition to protein synthesis, amino acids can be utilized for energy production, biosynthesis of nucleic acids and the synthesis of other macromolecules, such as fatty acids and nonessential amino acids. Recently, there has been considerable interest in the significant roles of amino acids as regulatory bioactive molecules involved in metabolic and energy homeostasis. Therefore, precise sensing of intracellular amino acid abundance is a highly important feature for efficiently coordinating proteins and other biosynthetic and catabolic pathways (Table 1). Enormous efforts have been made to identify intracellular amino acid sensors, many of which are associated and cooperate with mechanistic target of rapamycin complex 1 (mTORC1)^29^. mTORC1 is an important master effector that integrates nutrient signaling with cell growth and metabolism^30^. Because mTORC1 activity is modulated by specific sensors that are sensitive to fluctuations in amino acid levels, mTORC1 could be regarded as a signaling hub for amino acid metabolism and homeostasis (Fig. 2). Rag GTPases play essential roles as transducers of amino acid signaling for mTORC1 activation^31^. Four Rag GTPases assemble to form the heterodimers RagA-RagC and RagB-RagD. Translocation to lysosomes and activation of mTORC1 require GTP loading on RagA or RagC and GDP loading on RagB or RagD, and this modification of Rag GTPase heterodimers is modulated by amino acid signaling^32^.

**Table 1:** Amino acid sensors.

		Amino acid or surrogate sensed	Description	Reference
***mTORC1-related amino acid sensors***				
LARS1	Leucyl-tRNA synthetase 1	Leucine	Leucine-dependent GAP function for RagD and LARS1-Vps34-PLD1 axis activates mTORC1 under leucine stimulation.	^7,33,36,39,40^
Sestrin2	Sestrin2	Leucine	Leucine binding releases GATOR2 from Sestrin2, leading to mTORC1 activation.	^41–43^
SAMTOR	S-adenosylmethionine sensor upstream of TORC1	SAM	SAM binding dissociates SAMTOR from mTORC1, inhibiting the GATOR1–KICSTOR complex	^53–55^
CASTOR1	Cytosolic arginine sensor for MTORC1 Subunit 1	Arginine	Arginine binds to homodimeric CASTOR1 releases GATOR2 and leads to mTORC1 activation.	^60–62^
TM4SF5	Transmembrane 4 L six family member 5	Arginine	Interacts with mTOR and SLC38A9 in an arginine-dependent manner, promotes arginine efflux from lysosomes and activates mTORC1.	^66^
SLC38A9	Solute carrier family 38-member 9	Arginine	Lysosomal arginine sensing facilitates SLC38A9-mediated arginine efflux from lysosomal lumen.	^63–65^
TARS2	Threonyl-tRNA synthetase	Threonine	Threonine-bound TARS2 recruits GEFs and facilitates GTP loading of RagA and promotes mTORC1 activation.	^69^
***mTORC1-unrelated amino acid sensors***				
MARS1	Methionyl-tRNA synthetase	Methionine	Regulation of cell cycle via methionine-dependent interaction with CDK4.	^75^
QARS1	Glutaminyl-tRNA synthetase 1	Glutamine	QARS1 stabilizes ASK1 in inactive form and suppresses apoptotic signaling in a glutamine-dependent manner.	^82^
PAH	Phenylalanine hydroxylase	Phenylalanine	Allosteric binding of phenylalanine leads to conformational changes that enhance catalytic efficiency of PAH.	^84–86^
CARS1	Cysteinyl-tRNA synthetase 1	Cysteine	Absence of cysteine binding to CARS1 promotes its interaction with CaMKK2 and leads to AMPK activation and cell survival.	^89^
LCK	Lymphocyte-specific protein tyrosine kinase	Asparagine	Asparagine binding enhances LCK activity and leads to elevated T-cell activation and immune responses.	^90^
EPRS1	Glutamyl-prolyl-tRNA synthetase	Proline	EPRS1 mediates proline-dependent interaction with JAK1-STAT6-TGFβR1-SAMD3 during TGF-β-mediated induction of ECM components.	^91^
WARS1	Tryptophan-tRNA synthetase 1	Tryptophan	WARS1 interacts with PARP1 and DNA-PKcs under tryptophan deficiency and promotes p53 activation for anti-proliferative effects.	^92^
TDO	Tryptophan 2,3-dioxygenase	Tryptophan	High tryptophan levels stabilize TDO in the tetrameric structure for rapid degradation of excess dietary tryptophan.	^93,94^

#### mTORC1-related amino acid sensors

##### Leucine

**Fig. 3:**
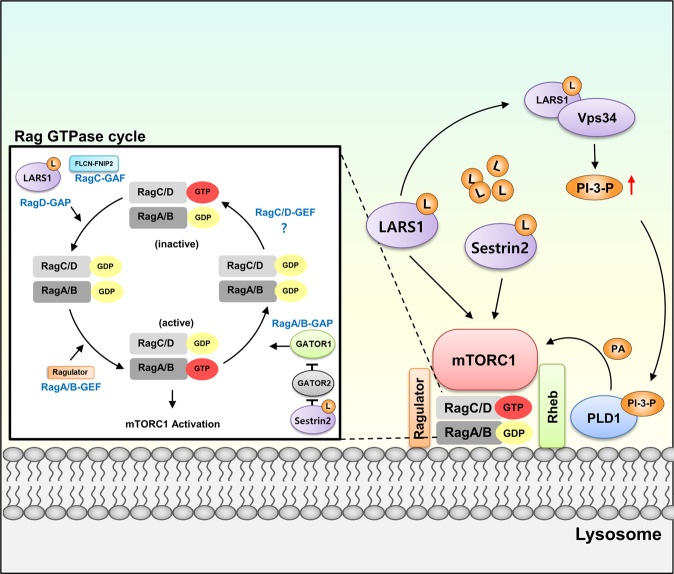
Leucine-sensing mechanism. Increased leucine concentration is sensed by different proteins to activate the mTOR pathway. When the leucine concentration is sufficiently high, LARS1 has leucine bound in the amino acid-binding site. This induces the activation of the leucine-dependent GTPase-activating protein function of LARS1. This hydrolysis of RagD-GTP results in RagD-GDP/RagB-GTP, which activates mTORC1. Leucine-bound LARS1 also interacts with Vps34. This activates the production of PI-3-P, which binds with PLD1 in the lysosome. PI-3-P-bound PLD1 activates mTORC1 through interaction with the Rheb protein. Sestrin2 also functions as a leucine sensor. Leucine-bound sestrin2 dissociates from GATOR2. Activated GATOR2 binds with GATOR1 to inhibit its function. The Rag GTPase negative regulator GATOR1 will end and activate RagA-GTP/RagC-GDP to increase the activity of mTORC1.

Leucine is a key amino acid that regulates the protein translation process of cells. It is also involved in food intake, muscle growth, and insulin secretion^33–35^. Leucine is thought to be an important amino acid because, compared with other amino acids, leucine is not metabolized at a high level^36^. This signifies that leucine levels are reflected via dietary consumption. Therefore, sensing the cellular leucine level is critical due to downstream signaling pathways that stimulate different cellular processes involving growth, including but not limited to protein synthesis, cellular growth, lipogenesis, and autophagy^37^. The leucine-sensing mechanism has been extensively researched (Fig. 3).

Leucyl-tRNA synthetase 1 (LARS1) was reported to function as a leucine sensor^7,38,39^. LARS1 is an enzyme that ligates tRNA to its cognate amino acid, leucine, by using a molecule of ATP that is consumed for protein synthesis in the translation process. Leucine can activate mTORC1 in a concentration-dependent manner^35^. Subsequently, its protein sensor function was investigated. After confirming that LARS1 is localized in the lysosomal membrane where mTORC1 becomes activated, the GTPase-activating protein function of LARS1 was discovered, as it forms a protein complex with RagD^7^. The leucine-sensing function of LARS1 was further verified by determining its leucine-dependent GAP function and lysosomal localization^40^. A higher leucine level allows the binding of leucine in the leucine binding site of LARS1. Then, leucine-bound LARS1 enables a conformational change that allows the binding of RagD, which induces RagD-GTP hydrolysis^41^. As a result, mTORC1 becomes activated, and downstream protein synthesis for cellular growth is initiated. An additional publication reported LARS1 as the leucine sensor for mTORC1 activation^42^. In response to leucine levels, the vacuolar protein sorting 34 (Vps34)-phospholipase D1 (PLD1) signaling pathway becomes activated. LARS1 is critical for this regulation, as leucine-bound LARS1 mechanically interacts with Vps34^42^.

Sestrin2 was discovered as another type of leucine sensor that activates the mTORC1 complex when leucine is bound to it^43^. When leucine levels are high, leucine will bind to sestrin2 at the suggested binding site at L261 and E451^44^. GATOR1 is a negative regulator of Rag GTPase activity by functioning as a GAP for RagA/B^45^. As leucine-bound sestrin2 inhibits GATOR2, the inhibition of GATOR1 by GATOR2 will halt^43^. This leads to activation of the RagA-GTP/RagC-GDP complex, which in turn stimulates Ragulator for mTORC1 activation^43^. However, the leucine sensory function of sestrin2 is still elusive due to its controversial function in different conditions^46–48^.

Later, the coordinating pathway between LARS1 and sestrin2 was discovered^49^. As both proteins were reported as leucine sensors for mTORC1 regulation, there were some questions as to how these two proteins work together. LARS1 and sestrin2 function as the ‘on’ and ‘off’ switches, respectively, in the RagD-RagB GTPase cycle^41,44^. Upon leucine availability, the inactive form of the RagD-RagB complex is activated by the RagD-GAP function by leucine-bound LARS1 and RagB-GEF function by Ragulator. The RagA-RagB GAP activity of GATOR1 is controlled by sestrin2, and it switches ‘off’ the Rag GTPase cycle. With the RagD-GEF function, the mechanism of which is still unknown, RagD-RagB returns to the inactive state. In the case of leucine sensing, different sensor proteins, LARS1 and sestrin2, coordinately function together to alter the RagD-RagB complex for mTORC1 activation upon leucine availability^49^. When LARS1 is chemically inhibited, its cellular leucine-sensing ability becomes damaged^50^. A chemical that binds to the RagD binding domain of LARS1 was reported in this research. This chemical was able to neutralize the leucine-sensing effect of LARS1, which led to mTORC1 inhibition irrespective of leucine concentration. A number of studies have confirmed that LARS1 effectively functions as a leucine sensor by directly interacting with leucine for downstream signaling of mTORC1 activation^51^. As leucine is an extremely important amino acid, the sensory function of LARS1 was researched as a therapeutic target for multiple diseases. Brain cancer, gastric cancer, kidney cancer, leukemia, myeloma, and sarcoma have high levels of LARS1 expression^8^. Therefore, LARS1 is targeted for anticancer treatment^52^.

##### Methionine

Methionine and its metabolites are essential for diverse cellular processes beyond their well-known roles in the initiation of translation. There are several mechanisms of intracellular methionine perception. For instance, an intracellular signal for methionine abundance is transduced to mTORC1 by SAMTOR, which serves as a sensor of S-adenosylmethionine (SAM) in the cytosol^53^. SAM is mainly synthesized from methionine by the reaction catalyzed by methionine adenosyltransferase (MAT), and it serves as a sole methyl group donor that participates in a wide range of biochemical processes, including nucleotide biosynthesis and epigenetic regulation^54^. SAM, as a surrogate marker for the intracellular abundance of methionine, is sensed to monitor the status of methionine metabolism^55^. Reduction in the intracellular methionine level is reflected as decreased SAM, which would allow the SAM-binding domain of SAMTOR to freely interact with GATOR1-KICSTOR^53,56,57^. Thus, methionine starvation leads to reduced SAM levels and promotes the association of SAMTOR with GATOR1-KICSTOR, which facilitates their inhibitory role on mTORC1 activity^53^. Conversely, when the SAM level is recovered, SAM binds to SAMTOR, leading to dissociation from GATOR1. This dissociation leads to the inactivation of GATOR1 and results in mTORC1 activation due to the prevention of GAP activity and increased GTP loading on RagA/B. Low intracellular levels of folate and cobalamin can lead to low SAM status; hence, SAMTOR could be considered a broad sensor^58^. SAMTOR coordinates methionine and one-carbon metabolism with mTORC1 signaling by sensing SAM.

##### Arginine

Arginine is another conditionally essential amino acid that participates in a variety of important metabolic pathways as the precursor of nitric oxide, polyamines, and creatine^59^. CASTOR1 binds directly with arginine in the cytosol at the defined binding pocket^60^. The arginine sensor CASTOR1 acts in a similar fashion as sestrin2. Upon arginine starvation, CASTOR1 binds and inhibits GATOR2 and thus suppresses mTORC1 activity^60,61^. GATOR2 is a positive regulator of mTORC1 that binds to GATOR1 and prohibits GATOR1-mediated suppression of mTORC1 activity^45^. Under arginine-abundant conditions, arginine binds to homodimeric CASTOR1 and induces conformational changes that facilitate its dissociation from GATOR2 and thus promote mTORC1 activation^62^. Therefore, CASTOR1 has been suggested to be a cytosolic arginine sensor that regulates mTORC1 activity according to intracellular arginine sufficiency.

In addition to the aforementioned cytosolic sensing machinery comprising CASTOR1, a lysosomal arginine-sensing branch also exists^63^. SLC38A9 is a transmembrane protein that mediates the efflux of arginine from the lysosomal lumen and is considered an important regulator in sensing lysosomal arginine levels^63,64^. However, the binding capacity for SLC38A9 remains unclear, and mTORC1 inhibition under arginine starvation is not reversed in human cells deficient in SLC38A9. Collectively, these contradictory results suggest that SLC38A9 might be less significant as a lysosomal arginine sensor^65^. In a later study, transmembrane 4 L six family member 5 (TM4SF5) was found to bind and interact with mTOR and SLC38A9 on lysosomal membranes under physiological arginine levels and possibly serve as a sensor of lysosomal arginine^66^. Under arginine sufficiency, the extracellular loop domain of TM4SF5 binds directly with arginine. Its translocation to the lysosomal membrane and binding with mTOR and SLC38A9 is promoted in an arginine-dependent manner, which leads to arginine efflux by SLC38A9 and subsequent mTOR/S6K1 activation^66^.

##### Threonine

Threonine is an essential amino acid that plays pivotal roles in the regulation of nutritional metabolism, cell growth, and proliferation^67^. Additionally, threonine exerts beneficial effects on lipid metabolism by modulating thermogenic gene expression and the lipogenesis pathway^68^. A recent study uncovered the role of the mitochondrial threonyl-tRNA synthetase TARS2 as the intracellular threonine sensor that is necessary for mTORC1 activation in a threonine-dependent manner^69^. In response to threonine stimulation, threonine-charged TARS2 interacts with inactive RagC and facilitates GTP loading of RagA by recruiting GEFs and subsequently allows Rags to recruit and activate mTORC1 by binding with Raptor. Furthermore, another report revealed that by activating mTORC1, TARS2 regulates cell proliferation and global mRNA translation^69^. TARS2 was reported to be overexpressed in many different types of cancer, and is especially highly activated in breast cancer^8^.

##### Glutamine

Glutamine is an important conditionally essential amino acid that is not only highly consumed as fuel for energy generation in highly proliferating cells but is also recognized as a key carbon and nitrogen source for diverse biosynthesis pathways. A more detailed review of the metabolic reliance of cells on glutamine is available elsewhere^70^. A study by Durán et al. demonstrated that alpha-ketoglutarate (αKG), synthesized from glutaminolysis, can activate mTORC1 by facilitating GTP loading of RagB; however, the underlying mechanism and the molecular sensor remain to be identified^71^. Glutamine also activates mTORC1 in a Rag-independent pathway^72^. Although the actual sensor to which glutamine binds has not yet been identified, in MEFs, adenosine diphosphate ribosylation factor-1 (Arf1) is required for glutamine-induced mTORC1 lysosomal localization and mTORC1 activation^73^. Arf1 was later found to activate the RalA-Rheb-PLD signaling apparatus to induce the activation of mTORC1^74^. However, Arf1-induced mTORC1 activation was also stimulated by asparagine^73^. Therefore, more research is required to understand whether Arf1 is a dual sensor.

#### mTORC1-unrelated Amino Acid Sensors

##### Methionine

Methionyl-tRNA synthetase (MARS1) is another intracellular methionine sensor that is linked to the regulation of the cell cycle^51^. MARS1 was demonstrated to interact with cyclin-dependent kinase 4 (CDK4) in a methionine-dependent manner^75^. Mechanistically, under sufficient or high methionine levels, MARS1 competes with the tumor suppressor p16^INK4a^ for interacting with CDK4 and augments complex formation and stabilization with CDK4, cell division cycle 37, HSP90 cochaperone (CDC37), and heat shock protein 90 (HSP90)^75^. However, under methionine-deficient conditions, MARS1 dissociates and favors CDK4 interaction with p16^INK4a^, which destabilizes the CDK4-HSP90-CDC37 complex and leads to subsequent ubiquitination and degradation of CDK4^76^. Therefore, MARS1- and CDK4-mediated cell cycle arrest ensures cellular and genomic integrity under methionine-restricted conditions. As MARS1 has been shown to be an important sensor, it is currently being studied as a biomarker and diagnostic marker for biliary diseases^77,78^.

##### Glutamine

Intracellular availability of glutamine is particularly important in cell survival and it is regarded as a suppressor of apoptotic cell death^79^. Many cancer cells utilize high contents of glutamine as metabolic fuel to support their proliferation, and the suppression of glutamine metabolism is considered an effective apoptosis-inducing therapeutic approach against cancer^80,81^. Intracellular glutamine sensing associated with apoptosis regulation is mediated by apoptosis signal-regulating kinase 1 (ASK1) and glutaminyl-tRNA synthetase 1 (QARS1)^82^. Glutamine binding to QARS1 promotes its association with ASK1, stabilizes ASK1 in its inactive form and suppresses apoptotic signaling. Under low-glutamine conditions, QARS1-mediated suppression of ASK1 is reduced, which allows for the autophosphorylation of ASK1. Therefore, QARS1 is an important intracellular glutamine sensor and key modulator of ASK1 that correlates glutamine abundance to apoptotic signals.

##### Phenylalanine

Phenylalanine is an essential amino acid that is recognized as the precursor for tyrosine and monoamine neurotransmitters, including dopamine, norepinephrine, and epinephrine. Although the cellular function of phenylalanine is poorly understood, the inability to metabolize phenylalanine due to the genetic loss of function of the enzyme phenylalanine hydroxylase (PAH) results in a serious disorder known as phenylketonuria (PKU)^83^. Apart from its crucial role in the catabolism of phenylalanine, PAH also plays an important role in sensing intracellular phenylalanine levels. Several studies have demonstrated that allosteric binding of phenylalanine alters the conformation of PAH and facilitates the formation of homotetrameric forms of PAH, which exhibit positive cooperativity toward L-phenylalanine^84–86^. Without allosteric binding of phenylalanine, the active site of phenylalanine is more occluded and favors homodimeric forms, which show reduced catalytic efficiency and act independently of phenylalanine concentrations^87^. Therefore, we can assume that autoregulation by PAH is followed by sensing of the intracellular phenylalanine supply and that conformational modification enhances the catalytic rates to fine-tune metabolic homeostasis.

##### Cysteine

Cysteine is a key substrate of glutathione biosynthesis. Therefore, it critically functions in the regulation of redox balance. Additionally, cysteine metabolism is important for coenzyme A and taurine synthesis^88^. A recent study demonstrated that under cysteine deficiency, cysteinyl-tRNA synthetase 1 (CARS1) promotes the binding of calcium/calmodulin-dependent protein kinase kinase 2 (CaMKK2) and AMPKγ2, which leads to the phosphorylation and activation of AMPK and cell survival under cysteine deprivation^89^. Cysteine binding to CARS1 inhibits the CaMKK2–CARS interaction to inactivate AMPK. Therefore, CARS1 is an important intracellular cysteine-specific sensor regulating AMPK activity that coordinates cysteine availability for cell survival. CARS1 has been highly researched for its secretion from cancer cells when activated by TNF-alpha. This increases the immune activation of macrophages^8^. The sensing mechanism of cysteine by CARS1 is correlated with immune activation in cancer.

##### Asparagine

T-cell activation induces amino acid transporter expression to upregulate amino acid uptake. In turn, intracellular amino acid availability is crucial for proper activation, proliferation and effector functions of T cells. In this regard, a recent discovery revealed that asparagine is particularly important for CD8^+^ T-cell activation and effector functions. In the same study, the SRC family protein tyrosine kinase LCK was found to serve as a core sensor signal to T cells during asparagine availability to T cells^90^. Mechanistically, direct binding of asparagine to LCK facilitates its phosphorylation at Tyr394 and Tyr505, which leads to enhanced LCK activity, T-cell activation, and immune responses. Conversely, restriction of asparagine to T cells led to impairment of LCK activity and T-cell activation.

##### Proline

Glutamyl-prolyl-tRNA synthetase (EPRS1) is a unique type of aminoacyl-tRNA synthetase that functions as a bifunctional protein comprising glutamyl-tRNA synthetase (ERS) and prolyl-tRNA synthetase (PRS)^91^. During TGF-β-mediated induction of ECM components for fibrogenesis, EPRS1 mediates the interaction of four different proteins, Janus kinases (JAKs), signal transducers and activators of transcription 6 (STAT6), TGF-β receptor 1 (TFGβR1), and mothers against decapentaplegic homolog 3 (SMAD3), in a proline-dependent manner. Low proline levels or blocking the catalytic site of EPRS1 by halofuginone abolishes this interaction and abrogates STAT6-dependent induction of ECM-related genes. Therefore, EPRS1 can modulate the generation of proline-rich ECM materials such as collagens by sensing intracellular proline availability^92^. EPRS1 is also highly expressed in cancer cells. The sensory function of EPRS1 is overly activated to maintain cancer cell survival^8^.

##### Tryptophan

The essential amino acid tryptophan is recognized for its important role as an immunomodulator. Upon immune cell activation by various insults against host organisms, the expression of indolamine 2,3-dioxidase (IDO) is greatly enhanced and rapidly depletes intracellular tryptophan. Deficiency of tryptophan leads to the translocation of tryptophan-tRNA synthetase 1 (WARS1) to the nucleus, where it forms a complex consisting of poly (ADP-ribose) polymerase 1 (PARP1) and the catalytic subunit of DNA-dependent protein kinase (DNA-PKcs). Subsequently, this complex activates the kinase function of DNA-PKcs by enhancing ADP-ribosylation, which promotes p53 activation and antiproliferative effects^93^. Thus, intracellular tryptophan availability is sensed by WARS1 to coordinate with cellular fate in proliferation by regulating p53 activity.

Alternatively, tryptophan 2,3-dioxygenase (TDO), which plays the same role as IDO and catalyzes the first rate-limiting step in tryptophan catabolism, is a tetrameric liver enzyme responsible for digestion of excess dietary tryptophan^94^. Recently, one group discovered that tryptophan consumption in the liver is controlled by TDO-mediated tryptophan sensing^95^. According to their findings, the stability of TDO is regulated in a tryptophan-dependent manner, where increasing tryptophan stabilizes the tetrameric structure of TDO for rapid degradation of tryptophan. However, under low-tryptophan conditions, TDO shifts to inactive monomers and dimers, which are susceptible to ubiquitin-mediated degradation^95^.

### Lipid sensing

Lipids comprise diverse sets of fatty acids (FAs) and sterols and are characterized by their hydrophobic carbon backbones. Lipids serve as the fundamental building blocks required for the generation of membranes and supplementation of energy and are important as a form of fuel storage. Furthermore, lipids function in regulating a wide range of biological processes, including gene expression and cell growth. Therefore, lipid biosynthesis is in high demand for rapidly growing cells. Lipids can serve as signals to control the cellular routes and capacity for their utilization, and the levels of lipid storage can be sensed by specific constituents in cells. Despite the increasing interest in intracellular lipid homeostasis, our understanding of lipid-sensing mechanisms, especially for fatty acids, is incomplete.

#### Cholesterol

Sterols are essential building blocks that confer membrane fluidity in mammalian membranes and utility in the biosynthesis of steroid hormones. De novo synthesis of cholesterol is tightly regulated because this pathway is energetically expensive, and it is possible to obtain cholesterol from the diet. Therefore, the ability to sense intracellular cholesterol is closely intertwined in the regulatory machineries in cholesterol biosynthetic pathways.

**Fig. 4:**
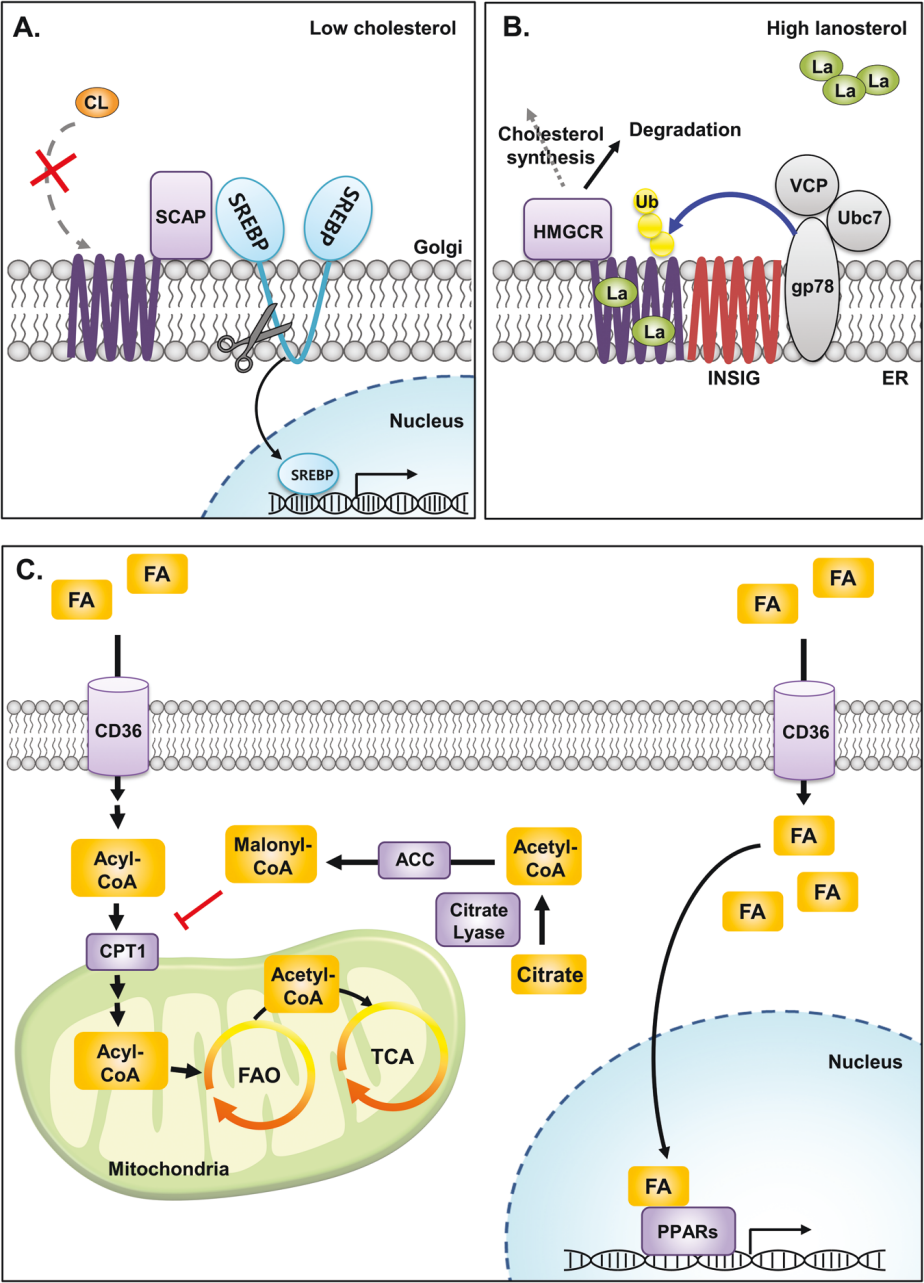
Lipid sensors. **A** Under cholesterol deprivation, cholesterol-unbound SCAP dissociates from INSIG (not shown), which favors SCAP-SREBP complex translocation from the ER to the Golgi. Subsequently, the cytoplasmic domain of SREBP is released by proteolytic cleavage, leading to the induction of key enzymes in cholesterol biosynthesis in the nucleus. **B** HMGCR is embedded in the ER and is responsible for the rate-limiting step in the de novo synthesis of cholesterol, which is especially important when low intracellular cholesterol levels are present. When intermediate sterol species, such as lanosterol, accumulate during the biosynthesis of cholesterol, HMGCR interacts with INSIG and the ubiquitination complex comprising VCP, gp78 and Ubc7. This interaction leads to the ubiquitination and subsequent degradation of HMGCR to immediately halt cholesterol synthesis. **C** Long-chain fatty acids are imported by membrane transporters, such as CD36. Fatty acids in the cytosol enter mitochondria by CPT1 on the outer membrane of mitochondria. CPT1 converts long chain acyl-CoA into acyl-carnitines, and this process is critical for FAO. Excess fatty acids synthesized by the cell are sensed by CPT1 via malonyl-CoA, the intermediate precursor of fatty acid synthesis, as the signal. Fluctuations in intracellular FAs can also be sensed by PPARs in the nucleus. Diverse species of FAs are capable of binding to the ligand binding domain in PPARs. Once activated, PPARs interact with various coactivators and induce the transcription of key regulatory genes in FAO and lipogenesis.

SREBP cleavage-activating protein (SCAP) is a membrane protein in the endoplasmic reticulum (ER) that functions as a cholesterol sensor and regulates the cholesterol content in mammalian cells^96^ (Fig. 4A). In the ER, SCAP is bound to the C-terminal extension of sterol regulatory element-binding proteins (SREBPs), which are the master transcription factors for key genes in cholesterol synthesis^97,98^. SCAP binds directly to cholesterol via its transmembrane sterol-sensing domains (SSDs), which reside in the lipid bilayer in the ER membrane^99^. When cholesterol is abundant, augmented cholesterol binding facilitates the conformational change in SCAP that enhances its affinity to INSIG, which acts as an anchor protein for SCAP-SREBP within ER membranes^99–101^. On the other hand, when intracellular cholesterol levels are decreased, cholesterol-free SCAP-SREBP detaches from INSIG and moves from the ER to the Golgi. Subsequently, proteases localized in the Golgi cleave the cytoplasmic amino-terminus of SREBP, which leads to the translocation of cleaved SREBP to the nucleus and allows the induction of genes for cholesterol biosynthesis^102,103^.

HMG-CoA reductase (HMGCR) is another key player in the cholesterol-sensing machinery in the ER. HMGCR catalyzes the rate-limiting step in the de novo cholesterol biosynthetic pathway and is also known to be the transcriptional target of SREBP under low cholesterol levels^104^. Apart from its catalytic domain, similar to SCAP, HMGCR also has a sterol-sensing domain embedded in the ER membrane. Increased levels of lanosterol, the intermediate product of cholesterol biosynthesis, promote the binding of HMGCR to INSIG^105^ (Fig. 4B). This binding facilitates HMGCR interaction with the ubiquitination complex comprising GP78, VCP and UBC, which leads to ubiquitin-mediated degradation of HMGCR^106^. This proteasome-dependent suppression of HMGCR allows rapid shutdown of energetically expensive cholesterol biosynthesis^104,107^. This important sensory function of HMGCR is currently applied to target dyslipidemia patients and clinically utilized.

#### Fatty Acids

The scavenger receptor CD36 is one of the most well-characterized fatty acid receptors/transporters that serves diverse functions in lipid signaling and metabolism^108^. By cooperating with other membrane proteins, CD36 governs the fatty acid uptake rate at the cell surface by directly binding to extracellular fatty acids^109^ (Fig. 4C). However, unlike its well-known role as a sensor for extracellular fatty acids, our current understanding regarding the role of CD36 in sensing fatty acid levels inside cells is limited. Nevertheless, several lines of evidence suggest that CD36 can coordinate intracellular fatty acid availability, reflecting dynamic metabolic demands. In addition to the cell membrane, CD36 is confined in intracellular compartments such as endosomes, and recent studies have revealed that CD36 plays a pivotal role in the regulation of cellular fatty acid uptake^108^.

Peroxisome proliferator-activated receptors (PPARs) are major transcriptional sensors of fatty acids that play critical roles in lipid homeostasis^110,111^. PPARs are ligand-activated transcription factors that belong to the nuclear receptor superfamily. Three PPAR subtypes show very distinctive tissue distributions and metabolic functions: PPARα (NR1C1), PPARβ or PPARδ (NR1C2), and PPARγ (NR1C3). PPARs are activated by a broad range of fatty acids and their derivatives, including unsaturated fatty acids, branched chain fatty acids, phospholipids, and oxidized and nitro-fatty acids. Upon binding of fatty acids in the ligand binding pocket in ligand-binding domains (LBD), conformational changes occur in the LBD that fix the C-terminal helix (helix 12) of the LBD in the ‘on’ position. This allows the interaction of the hydrophobic cavity with its coactivators through LXXLL motifs^112,113^. As a result, key regulatory genes involved in fatty acid oxidation (FAO) and/or lipogenesis are induced. For instance, recent studies have shown that nitro-fatty acids derived from the nitration of unsaturated fatty acids are agonists of PPARγ in human monocytes and macrophages. PPARγ activation by nitro-fatty acids leads to the upregulation of fatty acid binding protein 4 (FABP4), which in turn facilitates nitro-fatty acid-mediated downstream signaling^114^. Notably, apart from fatty acid binding-dependent activation, PPARs can be activated by ligand-independent signals, including cytokines and hormones, and participate in the regulation of inflammation, cellular growth, and differentiation. The role of PPAR as an intracellular fatty acid sensor is in part adaptable depending on the environmental conditions^110,115^.

Early studies proposed that carnitine palmitoyltransferase 1 (CPT1), a mitochondrial enzyme, has a rate-limiting role in intracellular fatty acid utilization^116^. CPT1 catalyzes the esterification of long chain acyl-CoA into acyl-carnitines to boost their entry into the mitochondria, which is the critical step for FAO. Although later studies questioned its role in the control of fatty acid uptake, CPT1 is still regarded as the regulator of mitochondrial β-oxidation^117,118^. Malonyl-CoA is the intermediate precursor of the fatty acid synthesis pathway and is produced from acetyl-CoA by the rate-limiting step catalyzed by acetyl-CoA carboxylase (ACC). Malonyl-CoA was first demonstrated to be capable of acting as an allosteric inhibitor of CPT1^119^. Under conditions of extensive fatty acid synthesis, accumulated malonyl-CoA blocks CPT1 activity to stop fatty acid β-oxidation and favors fatty acid biosynthesis. Therefore, malonyl-CoA is the key surrogate intermediate that is sensed by CPT1 to coordinate the catabolism and anabolism of fatty acids in a cell. Notably, the sensing of malonyl-CoA by CPT1 has been regarded as very important in the brain. Several studies have shown that this particular sensing mechanism is implicated in the control of food intake, weight loss, and other important biological functions in the regulation of the fate of neural stem cell precursors, development of axons, and metabolic coupling between neurons and astrocytes^120^.

## Crosstalk between nutrient sensors

### Crosstalk between glucose and amino acids

#### Leucine and glucose: O-GlcNAcylated LARS1, aldolase, and AMPK

**Fig. 5:**
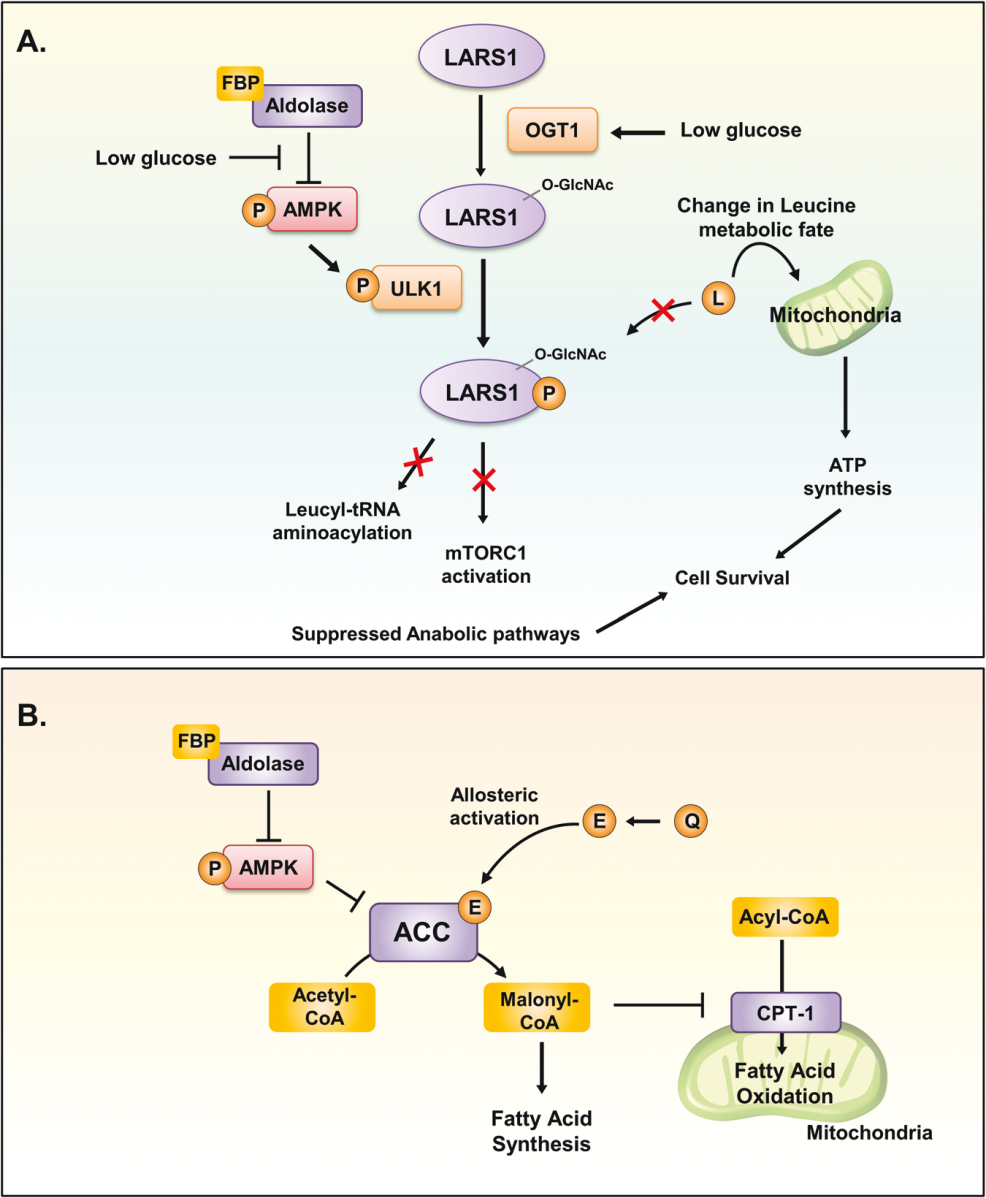
Crosstalk of nutrient sensors. **A** LARS1 and aldolase crosstalk with each other to control leucine and glucose availability, respectively. Under a limited-glucose environment, free aldolase allows the activation of AMPK, and OGT1 O-GlcNAcylates LARS1. AMPK phosphorylates ULK1. Activated ULK1 phosphorylates O-GlcNAc-LARS1. This inhibits the binding of leucine to LARS1 and modifies the fate of leucine to ATP synthesis instead of the activation of mTORC1 for protein synthesis. **B** Fatty acids and glucose are sensed by CPT1 and AMPK, respectively, for their crosstalk. Under nutrient supplementation, aldolase AMPK becomes inhibited, and as fatty acids are sufficiently supplemented, ACC activation is initiated by the allosteric activator citrate. ACC activation suppresses CPT1 function, which allows the production of malonyl-CoA to accumulate excess energy.

Currently, the crosstalk between leucine and glucose availability has been researched extensively. Leucine metabolism is differentially controlled depending on the glucose level^121^ (Fig. 5A). As AMPK is activated when glucose is deprived in a cell, AMPK will activate the kinase activity of ULK1. Then, ULK1 phosphorylates S720 of LARS1. The p-S720 LARS1 will not be able to bind leucine in its binding pocket, thus losing its leucine-sensing function. This will lead to increased concentrations of uncharged tRNA and denatured LARS1, leading to the inhibition of protein translation and mTORC1, respectively^41^. This research also proposed that nonbound leucine will undergo degradation to be utilized for ATP production in the mitochondria. Upon glucose starvation, LARS1 mediates the metabolic fate of leucine to ATP to support cell survival. Recently, direct evidence of glucose and leucine integration in the nutrient effector mTORC1 was revealed^122^. A specific posttranslational modification induced by glucose availability led to the modification of LARS1. O-GlcNAc modification is an enzymatic result of OGT1 function^123^. It uses a molecule of the glucose-derived metabolite N-acetylglucosamine to form a covalent bond on the oxygen atom of a protein^124^. Upon glucose limitation, OGT1 directs the posttranslational modification of LARS1 by forming an O-GlcNAcylated LARS1 on S1042. This directly blocks the interaction of LARS1 with RagD, and due to aldolase, AMPK is activated to phosphorylate S720 of O-GlcNAcylated LARS1. As a result, the cell will utilize leucine for energy production, and mTORC1 inhibition will lead to increased autophagy activity for cell survival under glucose-deprived conditions. This research was the first to introduce how two different nutrients could crosstalk for cellular survival and metabolism. Nutrients are hypothesized to have this kind of crosstalk ability to respond to different nutrient stresses as replacement nutrients must be utilized after the deprivation of other nutrients^125–127^. This research connected the glucose sensor aldolase and leucine sensor LARS1 with respect to their regulatory roles and exact mechanisms of fine-tuning metabolism and signaling pathways under glucose starvation stress.

### Crosstalk between glucose and lipids

#### Fatty acids and glucose: ACC, CPT1 and AMPK

The fatty acid metabolism pathway is another highly characterized target of AMPK. ACC is one of the well-characterized canonical targets of AMPK. Under stress conditions such as fasting, which lead to low-energy status, activated AMPK phosphorylates and inhibits ACC and reduces malonyl-CoA levels and fatty acid synthesis. Subsequently, FAO is unleashed to coordinate cell energy expenditure. The TCA cycle intermediate citrate is an allosteric activator of ACC. Under conditions of high glucose availability, citrate accumulates in the mitochondria and can be shuttled to the cytosol and converted to acetyl-CoA. During this process, citrate binds and allosterically activates ACC to increase malonyl-CoA production. This suppresses CPT1 activity and promotes fatty acid synthesis to store excess nutrients^128^. Therefore, glucose and fatty acid metabolic pathways are intertwined, and numerous mechanisms to coordinate anabolic and catabolic processes according to energy expenditure are available in cells.

The fatty acid sensor CPT1 was found to interact with AMPK activation^129^. This study suggested that glucose availability and fatty acid availability are integrated to control the activation of AMPK. This crosstalk was mainly researched in neuronal cells to understand the reaction of the hypothalamus in different nutritional states^129^. The same group demonstrated that during fasting, when both glucose and fatty acids are unavailable, AMPK and CPT1 both become active to increase hormone secretion to induce food intake^129^. AMPK becomes active when low glucose levels induce low FBP levels, which are sensed by aldolase, and CPT1 becomes active when low fatty acid levels induce low malonyl-CoA levels, which are sensed by CPT1^130^. The crosstalk between these two proteins results in the cellular exocytosis of hormones stimulating food intake.

### Crosstalk between amino acids and lipids

Amino acids can control plasma lipid levels. For instance, sulfur-containing amino acids such as cysteine and methionine are potent modulators of blood cholesterol^131^. Greater attention has been given to identifying the connections between lipid metabolism and amino acid metabolism; however, a large part of our understanding of the direct participation of the previously mentioned nutrient sensors in crosstalk remains to be uncovered.

#### Fatty acids and glutamine/glutamate: ACC

Glutamine/glutamate metabolism and fatty acid metabolism have been spotlighted due to their compensatory relationship. Under hypoxic conditions, glutamine/glutamate can act as an alternative supplier of citrate for fatty acid synthesis^132^. Reductive carboxylation of alpha-ketoglutarate derived from glutamine/glutamate generates citrate at a cost of NADPH by isocitrate dehydrogenase 2 (IDH2)^133,134^. Subsequently, citrate is shuttled out from mitochondria by citrate carrier (CIC) to be supplied in fatty acid synthesis.

Conversely, in the context of defective glutamine anaplerosis, upregulated fatty acid oxidation compensates for the energy expenditure of cancer cells as an alternative energy source^135^. In particular, glutaminase inhibitors induce cancer cell death by cutting off glutamine from the TCA cycle. However, metabolic reprogramming in fatty acid oxidation could occur and confer resistance to glutaminase inhibition^136^.

The interplay between glutamine/glutamate metabolism and fatty acid synthesis could be partly explained by the sensing of glutamate by the ACC (Fig. 5B). According to Boone et al., glutamate may function as an allosteric activator of the ACC upon binding^137^. Moreover, glutamate may have a complementary mode of action in the activation of the ACC by glutamate-sensitive protein phosphatase that dephosphorylates the ACC^137,138^. Therefore, glutamate sufficiency is also monitored by the ACC to mediate crosstalk between fatty acid synthesis and glutamine/glutamate metabolism to adjust metabolic status fit^139^. Thus, the ACC could accelerate fatty acid synthesis when support from glutamine/glutamate metabolism is granted. Unfortunately, other molecular sensors at the center of the crosstalk between glutamine/glutamate metabolism and lipid metabolism (i.e., fatty acid oxidation and cholesterol synthesis) remain elusive.

#### Cholesterol and leucine: LARS1, mTORC1, and FAF2

Recently, one group established a connection between mTORC1 and the lncRNA SNHG6^140^. Upon cholesterol availability, the FAF2 protein binds with cholesterol, leading to a conformational change for SNHG6 binding^141,142^. As SNHG6 binds to FAF2, mTORC1 lysosomal recruitment is increased, leading to its activation^140^. Although the authors did not experiment with different leucine concentrations, the leucine-dependent GTPase-activating protein function of LARS1 is critical in the initiation of mTORC1 activation. Therefore, this research suggests crosstalk between LARS1 and FAF2. Several studies have shown the importance of LARS1 in mTORC1 activation in various cell types, especially in cancer cells^143^. As the coupling effect of SNHG6 was investigated in cancer cells, cholesterol availability sensed by FAF2 was hypothesized to crosstalk with LARS1, which senses leucine availability, in turn activating mTORC1 for downstream signaling pathway activation in diseased states, such as cancer.

#### Fatty acids and amino acids: LARS1, mTORC1, mmBCFA

The crosstalk between fatty acids and amino acids has not been researched extensively. However, the relationship between fatty acids and amino acids can be examined from a developmental point of view. One group discovered the interaction between monomethyl branched-chain fatty acids (mmBCFAs) and leucine^144^. Normally, cells can produce different fatty acids and glycosphingolipids from metabolites derived from leucine molecules^145^. Therefore, leucine-derived monomethyl branched-chain fatty acids are directly connected. The actual sensor for mmBCFAs is still unknown, but it is hypothesized to directly activate mTORC1. This is because the level of mmBCFAs is decreased in the case of leucine deprivation, and mmBCFAs are important metabolites in the process of development^144^. For certain intestinal cells and adipocytes, the amount of mmBCFAs determines the distribution of peroxisomes, leading to different levels of autophagy, which demands a tight regulatory protein^146,147^. Although the actual interaction between these two sensors has yet to be discovered, LARS1 and the mmBCFA sensor could be suggested to interact directly to regulate the activation of mTORC1 and present different levels of autophagy.

## Conclusion

The mystery of the complete cellular signaling network has only been partially revealed. Nutrient sensing is one of the most important and basic signaling networks to be investigated^1^. Nutrient-sensing mechanisms are commonly undermined due to their effector functions and outcomes. Important phenotypes of the cells may be communicated by the nutrient sensor, but the cause and effect are more intensely researched^23,33,36^. Therefore, further studies on other independent amino acid sensors and lipid sensors should be conducted. Although the sensors for each amino acid and nutrient have not yet been discovered, the missing puzzle piece of the big picture remains to be found. There must be at least one sensor for each nutrient, and the downstream result will differ depending on the sufficiency of the nutrients. From an evolutionary perspective, aminoacyl-tRNA synthetase is a potential protein fulfilling this task^52^. However, much is still to be found. Due to the abundance of a nutrient, it will bind that molecule to the sensor for the cell to detect the richness of that nutrient and vice versa. Then, a nutrient signaling hub such as the mTORC1 complex will be controlled for the cell to respond. Leucine and glucose sensors are thoroughly understood, but the next step is to research the interactome of these sensors^7,36,122^. The crosstalk between different nutrients is not well understood. Numerous studies have shown only that crosstalk between nutrient sensors exists, but actual studies on the mechanism of crosstalk are lacking. Research on the mechanism will provide a better understanding of real cellular reactions. To illustrate, the leucine sensor may respond to leucine availability, but it is uncommon for a cell to be deprived of a single amino acid. The mechanisms underlying the interactions of sensors must be revealed to understand the complicated cellular signaling stimulated by various types of deprivation. There are still many excellent questions to be answered. Which nutrients interact with each other to respond to the nutrient status? Which nutrient sensor is the most powerful sensor for cellular survival? How are intracellular nutrient sensors cooperating with intercellular nutrient-sensing mechanisms? As the complex correlation between nutrient sensors is greatly implicated in human health, more questions need to be answered.
